# Supplementary material for: L-Ferritin: One Gene, Five Diseases; from Hereditary Hyperferritinemia to Hypoferritinemia—Report of New Cases
Source: Pharmaceuticals (Basel). 2019 Jan 23;12(1):17. doi: 10.3390/ph12010017 (PMC6469184; doi:10.3390/ph12010017)
Supplement: Supplementary file 1 [file pharmaceuticals-12-00017-s001.pdf]

SUPPLEMENTARY MATERIALS

L-Ferritin, one gene - five diseases; from hereditary hyperferritinaemia to hypoferritinaemia. Report of new cases.

Beatriz Cadenas [1,2,3], Josep Fita-Torró[4], Mar Bermúdez-Cortés [5], Inés Hernandez-Rodriguez [6], José Luis Fuster [5], María Esther Llinares[5], Ana María Galera[5], Julia Lee Romero [7], Santiago Pérez-Montero [4], Cristian Tornador [1,4], Mayka Sanchez [4,8,9]*

1 Whole Genix SL., Barcelona, Spain; Beatriz.cadenas@wholegenix.com
2 Iron Metabolism: Regulation and Diseases Group, Josep Carreras Leukaemia Research Institute (IJC), Campus Can Ruti, Badalona, Barcelona, Spain; bcadenas@carrerasresearch.org
3 Universitat de Vic-Universitat Central de Catalunya, Vic, Spain
4 BloodGenetics SL, Esplugues de Llobregat, Barcelona; Spain
5 Paediatric OncoHaematology Service, Clinic University Hospital Virgen de la Arrixaca. Instituto Murciano de Investigación Biosanitaria (IMIB), Murcia, Spain
6 Haematology Service, University Hospital Germans Trias i Pujol (HGTiP), Institut Català d'Oncologia (ICO), Badalona, Barcelona, Spain
7 University of Texas at Austin, Austin, Texas, United States
8 Program of Predictive and Personalised Medicine of Cancer (PMPPC), Institut d'Investigació Germans Trias i Pujol (IGTP), Campus Can Ruti, Badalona, Barcelona, Spain.
9 Iron metabolism: Regulation and Diseases Group, Faculty of Medicine and Health Sciences, Universitat Internacional de Catalunya (UIC), Barcelona, Spain.

* Correspondence: msanchezfe@uic.es; Tel.: + 34 935 042 000

Index

1. Supplementary Materials Table S1. Summary of *FTL* identified mutations.
2. Supplementary Materials Table S2. DNA sequence for *FTL* RNA fold predictions
3. Supplementary references

Supplementary Materials Table S1. Summary of *FTL* identified mutations.

Table summarizing all mutations in *FTL* described up to now in the literature that may cause one of the five diseases. The table shows for each mutation, the conventional nomenclature according to HGVS (corresponding to [NCBI:NM_000146.3] reference sequence), the traditional nomenclature, the position in the IRE structure, and the corresponding published report. NA (not available).

N	Enfermedad	HGVS nomenclature	Mutation type	Mutation position	First Publication	Old Nomenclature
1	**HHCS**	c.-216C>A	IRE regulatory	Promoter *FTL*	[1]	NA
2	HHCS	c.-193C>G + c.-160A>G	IRE regulatory	lower stem + hexanucleotide loop	[2]	+7C>G & +40A>G
3	HHCS	c.-190C>T	IRE regulatory	lower stem	[3]	+10C>U
4	HHCS	c.-186C>G	IRE regulatory	lower stem	[4]	+14C>G
5	HHCS	c.-184C>T	IRE regulatory	lower stem	[3]	+16C>U
6	HHCS	c.-182C>T + c.-178T>G	IRE regulatory	lower stem	[5]	Paiva-2 + 18C>U & 22U>G
7	HHCS	c.-176T>C	IRE regulatory	lower stem	[6]	+24U>C
8	HHCS	c.-171C>G	IRE regulatory	lower stem	[7]	Torino +29C>G
9	HHCS	c.-168G>A	IRE regulatory	lower stem	[5]	Pavia-1 +32G>A
10	HHCS	c.-168G>C	IRE regulatory	lower stem	[8]	Baltimore-1 +32G>C
11	HHCS	c.-168G>T	IRE regulatory	lower stem	[9]	Paris-2 or Milano-1 +32G>U
12	HHCS	c.-167C>A	IRE regulatory	C bulge	[10]	Paris +33C>A
13	HHCS	c.-167C>T	IRE regulatory	C bulge	[11]	Madrid or Philadelphia +33C>U
14	HHCS	c.-166T>C	IRE regulatory	upper stem	[12]	Paris +34U>C
15	HHCS	c.-164C>A	IRE regulatory	upper stem	[13]	London-2 +36C>A
16	HHCS	c.-164C>G	IRE regulatory	upper stem	[3]	Milano +36C>G
17	HHCS	c.-164C>T	IRE regulatory	upper stem	[14]	Badalona +36C>U
18	HHCS	c.-163A>C	IRE regulatory	upper stem	[15]	Pavia +37A>C
19	HHCS	c.-163A>G	IRE regulatory	upper stem	[3]	Milano +37A>G
20	HHCS	c.-163A>T	IRE regulatory	upper stem	[16]	Zaragoza +37A>U
21	HHCS	c.-161C>A	IRE regulatory	hexanucleotide loop	[17]	Geelong +39C>A

**22**	HHCS	c.-161C>G	IRE regulatory	hexanucleotide loop	[18]	Paris +39C>G
**23**	HHCS	c.-161C>T	IRE regulatory	hexanucleotide loop	[13]	London-1 +39C>U
**24**	HHCS	c.-160A>G	IRE regulatory	hexanucleotide loop	[19]	Paris-1 or Montpellier-1 +40A>G
**25**	HHCS	c.-160A>G + c.-159G>C	IRE regulatory	hexanucleotide loop	[4]	Paris-1 or Montpellier-1 +40A>G & Verona-1 +41G>C
**26**	HHCS	c.-159G>C	IRE regulatory	hexanucleotide loop	[20]	Verona-1 +41G>C
**27**	HHCS	c.-157G>A	IRE regulatory	hexanucleotide loop	[21]	Salt Lake City +43G>A
**28**	HHCS	c.-154T>G	IRE regulatory	upper stem	[22]	+46U>G
**29**	HHCS	c.-153G>A	IRE regulatory	upper stem	[12]	Paris +47G>A
**30**	HHCS	c.-151A>C	IRE regulatory	lower stem	[2]	+49A>C
**31**	HHCS	c.-151A>G	IRE regulatory	lower stem	[23]	Ghent +49A > G
**32**	HHCS	c.-150C>A	IRE regulatory	lower stem	[24]	+50C>A
**33**	HHCS	c.-149G>C	IRE regulatory	lower stem	[25]	Torino +51G>C
**34**	HHCS	c.-148G>C	IRE regulatory	lower stem	[14]	Heidelberg +52G>C
**35**	HHCS	c.-144A>T	IRE regulatory	lower stem	[15]	Paris +56A>U
**36**	HHCS	c.-110C>T	IRE regulatory	5′ UTR	[3]	+90C>U
**37**	HHCS	c.-220_-196del25	IRE regulatory	new transcription starting site (resulting IRE lacks nt 1-24)	[26]	NA
**38**	HHCS	c.-190-162del29	IRE regulatory	eliminating IRE	[27]	Verona-2 +10_38del29
**39**	HHCS	c.-182_-174delCGGGTCTGTinsAGGGGCCGG [$]	IRE regulatory	eliminating part of lower stem	[28]	+18_+26 delCGGGTCTGTinsAGGGGCCGG
**40**	HHCS	c.-178_-173del6	IRE regulatory	eliminating part of lower stem	[29]	+22_27del6
**41**	HHCS	c.-168_-165delGCTT	IRE regulatory	eliminating C bulge	[30]	+32_35delGCTT
**42**	HHCS	c.-164_158del7	IRE regulatory	eliminating part of	**THIS**	Esplugues +36_42del7

				hexanucleotide loop	**STUDY**	
**43**	HHCS	c.-161delC	IRE regulatory	eliminating IRE	[31]	+39delC
**44**	HHCS	c.-162_-161delCA	IRE regulatory	eliminating part of hexanucleotide loop	[32]	+38_39del AC
**45**	HHCS	c.-158_-143del16	IRE regulatory	eliminating part of hexanucleotide loop	[32]	+42_57del16
**46**	HHCS	c.-153_-152delGGinsCT	IRE regulatory	eliminating part of upper stem	[33]	+47_48delGGinsCT
**47**	HHCS	c.-44delT	IRE regulatory	eliminating IRE	[3]	
**1**	**Neuroferritinopathy**	c.[474G>A]; p.(Ala96Thr)	Missense	exon3	[34]	
**2**	Neuroferritinopathy	c.641_642 4bp_dup	Frameshift	exon 4	[35]	
**3**	Neuroferritinopathy	c.646InsC	Frameshift	exon 4	[36]	
**4**	Neuroferritinopathy	c.458dupA	Frameshift	exon 4	[37]	
**5**	Neuroferritinopathy	c.460InsA	Frameshift	exon 4	[38]	
**6**	Neuroferritinopathy	c.468_483 dup16nt	Frameshift	exon 4	[39]	
**7**	Neuroferritinopathy	c.469_484 dup16nt	Frameshift	exon 4	[40]	
**8**	Neuroferritinopathy	c.498InsTC	Frameshift	exon 4	[41]	
**9**	Neuroferritinopathy	c.468dupT	Frameshift	exon 4	[42]	
**10**	Neuroferritinopathy	c.467_470dupGTGG	Frameshift	exon 4	[43]	
**1**	**Beningn Hyperferritinaemia**	c.[77A<T]; p.(Gln26Leu)	Missense	exon 1	[44]	
**2**	Beningn Hyperferritinaemia	c.[80C>T]; p.(Ala27Val)	Missense	exon 1	[44]	
**3**	Beningn Hyperferritinaemia	c.[89C>T]; p.Thr30Ile	Missense	exon 1	[45]	
**1**	**L-ferritin deficiency Dominant**	c[1A>G]; p.(M1V)	Missense	start codon	[46]	
**2**	L-ferritin deficiency Dominant	c.375+2T>A	Splicing	intronic	**THIS STUDY**	
**1**	**L-ferritin deficiency**	c.[310G>T];p. (E104X)	Nonsense	exon3	[47]	

**Recesive**

NOTES:

[$]Previously reported by HGMD as c.-182_-176delCGGGTCTinsAGGGGCC, correct: c.-182_-174delCGGGTCTGTinsAGGGGCCGG

Supplementary Materials Table S2. DNA sequence for *FTL* RNA fold predictions

WT-IRE	5′-GCAGTTCGGCGGTCCCGCGGGTCTGTCTCTTGCTTCAACAGTGTTTGGACGGAACAGATCCGGGGACTCTCTTCC-3′
Mut-IRE	5′-GCAGTTCGGCGGTCCCGCGGGTCTGTCTCTTGCTTGTTTGGACGGAACAGATCCGGGGACTCTCTTCC-3′

Supplementary references

1. Faniello, M.C.; Di Sanzo, M.; Quaresima, B.; Nisticò, A.; Fregola, A.; Grosso, M.; Cuda, G.; Costanzo, F. Bilateral cataract in a subject carrying a C to A transition in the L ferritin promoter region. *Clin. Biochem.* **2009**, *42*, 911–914, doi:10.1016/j.clinbiochem.2009.02.013.
2. Castiglioni, E.; Soriani, N.; Girelli, D.; Camaschella, C.; Spiga, I.; Della Porta, M.G.; Ferrari, M.; Cremonesi, L. High resolution melting for the identification of mutations in the iron responsive element of the ferritin light chain gene. *Clin Chem Lab Med* **2010**, *48*, 1415–8, doi:10.1515/CCLM.2010.281.
3. Cremonesi, L.; Paroni, R.; Foglieni, B.; Galbiati, S.; Fermo, I.; Soriani, N.; Belloli, S.; Ruggeri, G.; Biasiotto, G.; Cazzola, M.; Ferrari, F.; Ferrari, M.; Arosio, P. Scanning mutations of the 5′UTR regulatory sequence of L-ferritin by denaturing high-performance liquid chromatography: identification of new mutations. *Br J Haematol* **2003**, *121*, 173–9.
4. Cremonesi, L.; Fumagalli, A.; Soriani, N.; Ferrari, M.; Levi, S.; Belloli, S.; Ruggeri, G.; Arosio, P. Double-gradient denaturing gradient gel electrophoresis assay for identification of L-ferritin iron-responsive element mutations responsible for hereditary hyperferritinemia-cataract syndrome: identification of the new mutation C14G. *Clin Chem* **2001**, *47*, 491–7.
5. Cazzola, M.; Bergamaschi, G.; Tonon, L.; Arbustini, E.; Grasso, M.; Vercesi, E.; Barosi, G.; Bianchi, P.E.; Cairo, G.; Arosio, P. Hereditary hyperferritinemia-cataract syndrome: relationship between phenotypes and specific mutations in the iron-responsive element of ferritin light-chain mRNA. *Blood* **1997**, *90*, 814–21.
6. Rufer, A.; Howell, J.P.; Lange, A.P.; Yamamoto, R.; Heuscher, J.; Gregor, M.; Wuillemin, W.A. Hereditary hyperferritinemia-cataract syndrome (HHCS) presenting with iron deficiency anemia associated with a new mutation in the iron responsive element of the L ferritin gene in a swiss family. *Eur J Haematol* **2011**, doi:10.1111/j.1600-0609.2011.01607.x.
7. Bosio, S.; Campanella, A.; Gramaglia, E.; Porporato, P.; Longo, F.; Cremonesi, L.; Levi, S.; Camaschella, C. C29G in the iron-responsive element of L-ferritin: a new mutation associated with hyperferritinemia-cataract. *Blood Cells. Mol. Dis.* **2004**, *33*, 31–34, doi:10.1016/j.bcmd.2004.04.010.
8. Kato, (first); Casella, F L-ferritin Baltimore-1: A novel mutation in the iron respinsive element (C32G) as a cause of hyperferritinemia-cataract syndrome. *Blood* **1999**, *94*, 407a.
9. Martin, M.E.; Fargion, S.; Brissot, P.; Pellat, B.; Beaumont, C. A point mutation in the bulge of the iron-responsive element of the L ferritin gene in two families with the hereditary hyperferritinemia-cataract syndrome. *Blood* **1998**, *91*, 319–23.
10. Durupt, S.; Durieu, I.; Salles, B.; Beaumont, C.; Hot, A.; Rousset, H.; Vital Durand, D. [A potential etiology of elevated ferritin: hyperferritinemia-cataract syndrome]. *Rev Med Interne* **2001**, *22*, 83–5.
11. Balas, A.; Aviles, M.J.; Garcia-Sanchez, F.; Vicario, J.L. Description of a new mutation in the L-ferrin iron-responsive element associated with hereditary hyperferritinemia-cataract syndrome in a Spanish family. *Blood* **1999**, *93*, 4020–1.
12. Hetet, G.; Devaux, I.; Soufir, N.; Grandchamp, B.; Beaumont, C. Molecular analyses of patients with hyperferritinemia and normal serum iron values reveal both L ferritin IRE and 3 new ferroportin (slc11A3) mutations. *Blood* **2003**, *102*, 1904–10, doi:10.1182/blood-2003-02-0439.
13. Mumford, A.D.; Vulliamy, T.; Lindsay, J.; Watson, A. Hereditary hyperferritinemia-cataract syndrome: two novel mutations in the L-ferritin iron-responsive element. *Blood* **1998**, *91*, 367–8.
14. Luscieti, S.; Tolle, G.; Aranda, J.; Campos, C.B.; Risse, F.; Morán, É.; Muckenthaler, M.U.; Sánchez, M. Novel mutations in the ferritin-L iron-responsive element that only mildly impair IRP binding cause hereditary hyperferritinaemia cataract syndrome. *Orphanet J. Rare Dis.* **2013**, *8*, 30, doi:10.1186/1750-1172-8-30.
15. Ferrari, F.; Foglieni, B.; Arosio, P.; Camaschella, C.; Daraio, F.; Levi, S.; García Erce, J.A.; Beaumont, C.; Cazzola, M.; Ferrari, M.; Cremonesi, L. Microelectronic DNA chip for hereditary hyperferritinemia cataract syndrome, a model for large-scale analysis of disorders of iron metabolism. *Hum. Mutat.* **2006**, *27*, 201–208, doi:10.1002/humu.20294.
16. Garcia Erce, J.A.; Cortes, T.; Cremonesi, L.; Cazzola, M.; Perez-Lungmus, G.; Giralt, M. [Hyperferritinemia-cataract syndrome associated to the HFE gene mutation. Two new Spanish families and a new mutation (A37T: "Zaragoza")]. *Med Clin Barc* **2006**, *127*, 55–8.
17. McLeod, J.L.; Craig, J.; Gumley, S.; Roberts, S.; Kirkland, M.A. Mutation spectrum in Australian pedigrees with hereditary hyperferritinaemia-cataract syndrome reveals novel and de novo mutations. *Br J Haematol* **2002**, *118*, 1179–82.

18. Garderet, L.; Hermelin, B.; Gorin, N.C.; Rosmorduc, O. Hereditary hyperferritinemia-cataract syndrome: a novel mutation in the iron-responsive element of the L-ferritin gene in a French family. *Am J Med* **2004**, *117*, 138–9, doi:10.1016/j.amjmed.2004.02.033.
19. Beaumont, C.; Leneuve, P.; Devaux, I.; Scoazec, J.Y.; Berthier, M.; Loiseau, M.N.; Grandchamp, B.; Bonneau, D. Mutation in the iron responsive element of the L ferritin mRNA in a family with dominant hyperferritinaemia and cataract. *Nat Genet* **1995**, *11*, 444–6, doi:10.1038/ng1295-444.
20. Meneses, F.G.A.; Schnabel, B.; Silva, I.D.C.G.; Alberto, F.L.; Toma, L.; Nader, H.B.; Lopes, C.C. Identification of the mutations associated with hereditary hyperferritinemia cataract syndrome and hemochromatosis in a Brazilian family. *Clin. Genet.* **2011**, *79*, 189–192, doi:10.1111/j.1399-0004.2010.01517.x.
21. Phillips, J.D.; Warby, C.A.; Kushner, J.P. Identification of a novel mutation in the L-ferritin IRE leading to hereditary hyperferritinemia-cataract syndrome. *Am J Med Genet A* **2005**, *134A*, 77–9, doi:10.1002/ajmg.a.30425.
22. Messa, E.; Pellegrino, R.M.; Palmieri, A.; Carturan, S.; Cilloni, D.; Saglio, G.; Roetto, A. Identification of a novel mutation in the L ferritin iron-responsive element causing hereditary hyperferritinemia-cataract syndrome. *Acta Haematol* **2009**, *122*, 223–5, doi:10.1159/000253031.
23. Sompele, S.V. de; Pécheux, L.; Couso, J.; Meunier, A.; Sanchez, M.; Baere, E.D. Functional characterization of a novel non - coding mutation "Ghent +49A > G" in the iron-responsive element of L-ferritin causing hereditary hyperferritinaemia-cataract syndrome. *Sci. Rep.* **2017**, *7*, 18025, doi:10.1038/s41598-017-18326-6.
24. Gonzalez-Huerta, L.; Ramirez-Sanchez, V.; Rivera-Vega, M.; Messina-Baas, O.; Cuevas-Covarrubias, S. A family with hereditary hyperferritinaemia cataract syndrome: evidence of incomplete penetrance and clinical heterogeneity. *Br J Haematol* **2008**, *143*, 596–8, doi:10.1111/j.1365-2141.2008.07345.x.
25. Camaschella, C.; Zecchina, G.; Lockitch, G.; Roetto, A.; Campanella, A.; Arosio, P.; Levi, S. A new mutation (G51C) in the iron-responsive element (IRE) of L-ferritin associated with hyperferritinaemia-cataract syndrome decreases the binding affinity of the mutated IRE for iron-regulatory proteins. *Br. J. Haematol.* **2000**, *108*, 480–482.
26. Burdon, K.P.; Sharma, S.; Chen, C.S.; Dimasi, D.P.; Mackey, D.A.; Craig, J.E. A novel deletion in the FTL gene causes hereditary hyperferritinemia cataract syndrome (HHCS) by alteration of the transcription start site. *Hum Mutat* **2007**, *28*, 742, doi:10.1002/humu.9501.
27. Girelli, D.; Corrocher, R.; Bisceglia, L.; Olivieri, O.; Zelante, L.; Panozzo, G.; Gasparini, P. Hereditary hyperferritinemia-cataract syndrome caused by a 29-base pair deletion in the iron responsive element of ferritin L-subunit gene. *Blood* **1997**, *90*, 2084–8.
28. Lenzhofer, M.; Schroedl, F.; Trost, A.; Kaser-Eichberger, A.; Wiedemann, H.; Strohmaier, C.; Hohensinn, M.; Strasser, M.; Muckenthaler, M.U.; Grabner, G.; Aigner, E.; Reitsamer, H.A. Aqueous humor ferritin in hereditary hyperferritinemia cataract syndrome. *Optom Vis Sci* **2015**, *92*, S40-7, doi:10.1097/OPX.0000000000000544.
29. Cazzola, M.; Foglieni, B.; Bergamaschi, G.; Levi, S.; Lazzarino, M.; Arosio, P. A novel deletion of the L-ferritin iron-responsive element responsible for severe hereditary hyperferritinaemia-cataract syndrome. *Br J Haematol* **2002**, *116*, 667–70.
30. Garber, I.; Pudek, M. A novel deletion in the iron-response element of the L-ferritin gene, causing hyperferritinaemia cataract syndrome. *Ann. Clin. Biochem.* **2014**, *51*, 710–713, doi:10.1177/0004563214542289.
31. Muñoz-Muñoz, J.; Cuadrado-Grande, N.; Moreno-Carralero, M.-I.; Hoyos-Sanabria, B.; Manubes-Guarch, A.; González, A.-F.; Tejada-Palacios, P.; Del-Castillo-Rueda, A.; Morán-Jiménez, M.-J. Hereditary hyperferritinemia cataract syndrome in four patients with mutations in the IRE of the FTL gene. *Clin. Genet.* **2013**, *83*, 491–493, doi:10.1111/j.1399-0004.2012.01934.x.
32. Giansily, M.; Beaumont, C.; Desveaux, C.; Hetet, G.; Schved, J.F.; Aguilar-Martinez, P. Denaturing gradient gel electrophoresis screening for mutations in the hereditary hyperferritinaemia cataract syndrome. *Br J Haematol* **2001**, *112*, 51–4.
33. Mattila, R.M.; Sainio, A.; Jarvelainen, M.; Pursiheimo, J.; Jarvelainen, H. A novel double nucleotide variant in the ferritin-L iron-responsive element in a Finnish patient with hereditary hyperferritinaemia-cataract syndrome. *Acta Ophthalmol* **2018**, *96*, 95–99, doi:10.1111/aos.13492.
34. Maciel, P.; Cruz, V.T.; Constante, M.; Iniesta, I.; Costa, M.C.; Gallati, S.; Sousa, N.; Sequeiros, J.; Coutinho, P.; Santos, M.M. Neuroferritinopathy: Missense mutation in FTL causing early-onset bilateral pallidal involvement. *Neurology* **2005**, *65*, 603–605, doi:10.1212/01.wnl.0000178224.81169.c2.
35. Kubota, A.; Hida, A.; Ichikawa, Y.; Momose, Y.; Goto, J.; Igeta, Y.; Hashida, H.; Yoshida, K.; Ikeda, S.-I.; Kanazawa, I.; Tsuji, S. A novel ferritin light chain gene mutation in a Japanese family with neuroferritinopathy: description of clinical features and implications for genotype-phenotype correlations. *Mov. Disord. Off. J. Mov. Disord. Soc.* **2009**, *24*, 441–445, doi:10.1002/mds.22435.

36. Mancuso, M.; Davidzon, G.; Kurlan, R.M.; Tawil, R.; Bonilla, E.; Di Mauro, S.; Powers, J.M. Hereditary ferritinopathy: a novel mutation, its cellular pathology, and pathogenetic insights. *J. Neuropathol. Exp. Neurol.* **2005**, *64*, 280–294.
37. Devos, D.; Tchofo, P.J.; Vuillaume, I.; Destée, A.; Batey, S.; Burn, J.; Chinnery, P.F. Clinical features and natural history of neuroferritinopathy caused by the 458dupA FTL mutation. *Brain* **2009**, *132*, e109, doi:10.1093/brain/awn274.
38. Curtis, A.R.; Fey, C.; Morris, C.M.; Bindoff, L.A.; Ince, P.G.; Chinnery, P.F.; Coulthard, A.; Jackson, M.J.; Jackson, A.P.; McHale, D.P.; Hay, D.; Barker, W.A.; Markham, A.F.; Bates, D.; Curtis, A.; Burn, J. Mutation in the gene encoding ferritin light polypeptide causes dominant adult-onset basal ganglia disease. *Nat. Genet.* **2001**, *28*, 350–354, doi:10.1038/ng571.
39. Nishida, K.; Garringer, H.J.; Futamura, N.; Funakawa, I.; Jinnai, K.; Vidal, R.; Takao, M. A novel ferritin light chain mutation in neuroferritinopathy with an atypical presentation. *J. Neurol. Sci.* **2014**, *342*, 173–177, doi:10.1016/j.jns.2014.03.060.
40. Ohta, E.; Nagasaka, T.; Shindo, K.; Toma, S.; Nagasaka, K.; Ohta, K.; Shiozawa, Z. Neuroferritinopathy in a Japanese family with a duplication in the ferritin light chain gene. *Neurology* **2008**, *70*, 1493–1494, doi:10.1212/01.wnl.0000310428.74624.95.
41. Vidal, R.; Ghetti, B.; Takao, M.; Brefel-Courbon, C.; Uro-Coste, E.; Glazier, B.S.; Siani, V.; Benson, M.D.; Calvas, P.; Miravalle, L.; Rascol, O.; Delisle, M.B. Intracellular ferritin accumulation in neural and extraneural tissue characterizes a neurodegenerative disease associated with a mutation in the ferritin light polypeptide gene. *J. Neuropathol. Exp. Neurol.* **2004**, *63*, 363–380.
42. Moutton, S.; Fergelot, P.; Trocello, J.-M.; Plante-Bordeneuve, V.; Houcinat, N.; Wenisch, E.; Larue, V.; Brugières, P.; Clot, F.; Lacombe, D.; Arveiler, B.; Goizet, C. A novel FTL mutation responsible for neuroferritinopathy with asymmetric clinical features and brain anomalies. *Parkinsonism Relat. Disord.* **2014**, *20*, 935–937, doi:10.1016/j.parkreldis.2014.04.026.
43. Ni, W.; Li, H.-F.; Zheng, Y.-C.; Wu, Z.-Y. FTL mutation in a Chinese pedigree with neuroferritinopathy. *Neurol. Genet.* **2016**, *2*, e74, doi:10.1212/NXG.0000000000000074.
44. Thurlow, V.; Vadher, B.; Bomford, A.; DeLord, C.; Kannengiesser, C.; Beaumont, C.; Grandchamp, B. Two novel mutations in the L ferritin coding sequence associated with benign hyperferritinaemia unmasked by glycosylated ferritin assay. *Ann. Clin. Biochem.* **2012**, *49*, 302–305, doi:10.1258/acb.2011.011229.
45. Kannengiesser, C.; Jouanolle, A.M.; Hetet, G.; Mosser, A.; Muzeau, F.; Henry, D.; Bardou-Jacquet, E.; Mornet, M.; Brissot, P.; Deugnier, Y.; Grandchamp, B.; Beaumont, C. A new missense mutation in the L ferritin coding sequence associated with elevated levels of glycosylated ferritin in serum and absence of iron overload. *Haematologica* **2009**, *94*, 335–9, doi:10.3324/haematol.2008.000125.
46. Cremonesi, L.; Cozzi, A.; Girelli, D.; Ferrari, F.; Fermo, I.; Foglieni, B.; Levi, S.; Bozzini, C.; Camparini, M.; Ferrari, M.; Arosio, P. Case report: a subject with a mutation in the ATG start codon of L-ferritin has no haematological or neurological symptoms. *J. Med. Genet.* **2004**, *41*, e81.
47. Cozzi, A.; Santambrogio, P.; Privitera, D.; Broccoli, V.; Rotundo, L.I.; Garavaglia, B.; Benz, R.; Altamura, S.; Goede, J.S.; Muckenthaler, M.U.; Levi, S. Human L-ferritin deficiency is characterized by idiopathic generalized seizures and atypical restless leg syndrome. *J. Exp. Med.* **2013**, *210*, 1779–1791, doi:10.1084/jem.20130315.
